# Sentiment Analysis of Arabic Tweets Regarding Distance Learning in Saudi Arabia during the COVID-19 Pandemic

**DOI:** 10.3390/s21165431

**Published:** 2021-08-11

**Authors:** Malak Aljabri, Sara Mhd. Bachar Chrouf, Norah A. Alzahrani, Leena Alghamdi, Reem Alfehaid, Reem Alqarawi, Jawaher Alhuthayfi, Nouf Alduhailan

**Affiliations:** 1 Computer Science Department, College of Computer and Information Science, Umm AlQura University, Makkah 21961, Saudi Arabia; 2 Department of Computer Science, College of Computer Science and Information Technology, Imam Abdulrahman Bin Faisal University, Dammam 31441, Saudi Arabia; 2170007790@iau.edu.sa (S.M.B.C.); 2170005400@iau.edu.sa (N.A.A.); 2170006635@iau.edu.sa (L.A.); 2170005031@iau.edu.sa (R.A.); 2170006008@iau.edu.sa (R.A.); 2170005195@iau.edu.sa (J.A.); 2170002706@iau.edu.sa (N.A.)

**Keywords:** COVID-19, distance learning, Twitter, sentiment analysis

## Abstract

The COVID-19 pandemic has greatly impacted the normal life of people worldwide. One of the most noticeable impacts is the enforcement of social distancing to reduce the spread of the virus. The Ministry of Education in Saudi Arabia implemented social distancing measures by enforcing distance learning at all educational stages. This measure brought about new experiences and challenges to students, parents, and teachers. This research measures the acceptance rate of this way of learning by analysing people’s tweets regarding distance learning in Saudi Arabia. All the tweets analysed were written in Arabic and collected within the boundary of Saudi Arabia. They date back to the day that the distance learning announcement was made. The tweets were pre-processed, and labelled positive, or negative. Machine learning classifiers with different features and extraction techniques were then built to analyse the sentiment. The accuracy results for the different models were then compared. The best accuracy achieved (0.899) resulted from the Logistic regression classifier with unigram and Term Frequency-Inverse Document Frequency as a feature extraction approach. This model was then applied on a new unlabelled dataset and classified to different educational stages; results demonstrated generally positive opinions regarding distance learning for general education stages (kindergarten, intermediate, and high schools), and negative opinions for the university stage. Further analysis was applied to identify the main topics related to the positive and negative sentiment. This result can be used by the Ministry of Education to further improve the distance learning educational system.

## 1. Introduction

The coronavirus that caused a worldwide pandemic, known as COVID-19, was first reported in December 2019 in Wuhan City, Hubei Province of China. COVID-19 causes a respiratory illness, ranging from common flu symptoms to severe illness and even death in some cases. As of January 2021, over 100 million cases have been reported around the world, with a mortality rate of around 2% of the total closed cases [1,2]. This rapidly expanding pandemic is of global concern and presents a serious threat to public health and the economy worldwide.

Countries around the world have applied various prevention measures to restrict the spread of the virus. An important prevention measure applied in most countries was the temporary closure of schools and universities. Distance learning was introduced instead. On 8 March 2020, the Ministry of Education in Saudi Arabia enforced distance learning for all school and university students. The Ministry already had a system that featured smart classrooms and education management systems, where a student can acquire textbooks online and take online assessments instead of physically sitting for exams, which proved useful during the emergence of the COVID-19 pandemic [3].

Despite the Ministry’s great efforts to facilitate distance learning, various challenges surfaced due to the sudden transition, including unreliable internet connections and limited computers or smart devices for some people; difficulty to adapt to distance learning technologies, especially by primary school students and their parents; limited infrastructure capacity; and, difficulties connected with accurately measuring students’ academic performance through distance learning [4].

Artificial Intelligence (AI) has transformed our daily lives in unbelievable ways. It has become significantly important as it tackles a profound set of technical challenges. Sentiment Analysis is a specific technique of Machine Learning (ML), one important branch of AI. It has become an essential technology to use in data analytics as it extracts insights from text. This is done by analyzing the ever-expanding datasets collected from different sources, such as people’s posts on social media, to obtain an overview of public opinion on specific topics. The applications of sentiment analysis are powerful. Companies around the world are increasingly using sentiment analysis techniques to gather insight into their customers’ opinions about their products or services based on their posts on social media. This type of feedback greatly assists companies in making future decisions and improving their services.

The main goal of this research is to conduct sentiment analysis studies to analyze opinions on the introduction of distance learning in Saudi Arabia as one of the main prevention measures to restrict the spread of COVID-19. Analyzing people’s opinions can help the Ministry of Education visualize how people are coping with the situation. This awareness can help the Ministry to improve distance learning technologies in schools and universities and make the experience more productive. The goal of this research was achieved by collecting an Arabic tweets dataset from Twitter using specific keywords that focused on distance learning at different educational stages (university, intermediate/high schools, and primary schools/kindergarten) and then applying different pre-processing and features extraction techniques to prepare the collected tweets for analysis. Different machine learning approaches were then employed to analyze the tweets. The different approaches were then measured and compared. Finally, the best model was applied on new a dataset classified to different educational stages to draw conclusions about the distance learning acceptance rate.

Sentiment analysis is increasingly conducted in literature, especially with the increase in social data being generated. However, most of the research activities reported in the literature concerning sentiment analysis concern data generated in the English language more than any other language.

In the following paragraphs we present some studies that are relevant to this research. Specifically, research that applied ML sentiment analysis to Arabic tweets to measure people’s opinions on different topics. All the studies highlighted here applied similar techniques in terms of pre-processing. Specifically, they all followed the same basics steps for cleaning tweets which include removing unrelated data and special symbols, stop words filtering, applying normalisation, etc. In the discussion of these relevant studies, the focus is on the number of tweets analysed, the number of categories used to classify tweets, the machine learning algorithm applied, and the level of accuracy achieved.

With the goal of identifying people’s opinions on health services, [5] collected and analysed a dataset of 2026 Arabic tweets. They annotated the tweets to either a positive or negative category and applied a combination of unigram and bigram techniques with the TF-IDF method to weigh each feature in the corpus. The top 1000 maximum weighted features were then fed into the machine learning algorithm. For the classification process, the best accuracy results achieved (up to 91%) were with the Support Vector Machine (SVM) using the Linear Support Vector Classification and Stochastic Gradient Descent (SGD).

Using a similar dataset size, but with a different goal and classification model, [6] applied sentiment analysis on a dataset of 2000 Arabic tweets about the Syrian civil war and crisis. The tweets (that did not exceed 140 characters in length) were collected in 2014. They annotated the tweets to either positive or negative category, and the Information Gain measure was applied to extract and select features. The researchers used machine learning algorithms and a lexicon-based approach for sentiment prediction and then compared their performance. In the lexicon-based approach, the researchers used the Arabic Emoticon Lexicon to classify each tweet, expressed as a bag-of-words (BOW), according to two labels: positive or negative. This was done through a lookup process, where every word in the tweet is looked up in the Arabic Lexicon. If the word is not included in the lexicon, it is treated as a neutral word and given a score of zero. The tweet’s overall sentiment score was then calculated, and based on this calculation, the tweet was given a sentiment label that was either positive or negative. For the machine learning approach, the researchers used different algorithms to classify the tweets. The experiment showed that the approach based on the use of machine learning algorithms performed better than the approach that was based on the use of the lexicon in predicting the subjectivity of tweets. In terms of machine learning algorithms, the Logistic Model Trees (LMT) algorithm reached the highest accuracy results of 85.5%, using the top 150 features.

In another work, [7] applied machine learning techniques to capture online hate speech in a dataset of 1633 Arabic tweets. Each tweet was labelled positive if it represented hate speech and negative if it represented non-hate speech. For feature extraction, the researchers also applied a combination of BoW and TF-IDF. It is worth mentioning that, in this study, as well as word features, the researchers considered emotion features and profile-related features such as ‘retweet-count’ and ‘favourite-count’. Several machine learning algorithms were used for the classification, and it was concluded that the highest performance was achieved when using Random Forest (RF), with an accuracy of 91%.

Al-Rubaiee et al. [8] carried out a study to classify Arabic tweets according to whether they featured a positive, negative, or neutral opinion about e-learning. They started by collecting tweets and then they performed pre-processing, which resulted in 1121 tweets. They then manually labelled the tweets positive, negative, or neutral. They applied SVM and Naïve Bayes (NB) to classify the tweets, using the TF-IDF weighting scheme along with N-grams for features extraction. They then carried out two experiments with different classes. The first experiment comprised positive and negative categories, while the second experiment comprised positive, negative, and neutral categories. The best performing model was the SVM that achieved an accuracy of 84.84% in the first experiment, and 73.15% in the second.

The method described by [9] used Arabic Twitter data to explore depressive emotions in an online population from the Gulf region. The researchers initially obtained tweets from 97 users, 35 of whom were depressed. After pre-processing, the researchers manually annotated the remaining data, consisting of 6122 tweets, according to depression scale tools. Out of the 6122 tweets, 1359 tweets were labelled depressed, 1363 non-depressed, and 3400 labelled ‘other’ and then eliminated. The BoW was subsequently applied as a feature extraction with negation handling, as the negation was an important factor in analyzing the sentiment of the tweets (e.g., not happy). Thus, the researchers applied some modifications in the filter to make it compatible with Arabic tweets by adding a list of Arabic negation words. Finally, they used Weka to carry out supervised machine learning using four classifiers: Random Forest, Naive Bayes, AdaBoostM1, and Liblinear [10], which is an open-source package that supports linear SVM and provides efficient performance for large scale classification. Their results indicated that the Liblinear had higher accuracy than other classifiers, with 87.5% accuracy.

All the above studies followed similar methodologies that differed only in the details relating to feature extraction techniques and the applied machine learning models. However, compared to this study, they all used a small dataset (of around 2000 tweets).

The study that is most closely related to this research work is a recent study conducted by Alhajji et al. [11] to analyse sentiment regarding the key preventive measures that were implemented following confirmation of the first COVID-19 cases in Saudi Arabia. The study focused on analysing tweets generated from 5–23 March relating to the following seven key decisions:

1. Holy Mosque closure for certain times daily for sterilization purposes.
2. Traffic Restriction to/from Qatif city, where the first COVID-19 confirmed cases were registered.
3. Closure of education centres including schools and universities across the Kingdom and switching to distance learning.
4. Closure of most of the services and places including parks and shopping centres, apart from some important services such as pharmacies and large grocery shops.
5. Suspension of all sports activities and competitions.
6. Suspension of the prayers in all mosques across the Kingdom.
7. Starting the curfew across the Kingdom for 21 days.

The method followed to achieve the main goal of the study included several steps, starting from downloading related tweets within 48 h of the announcement of each prevention measure, then pre-processing the collected tweets. Unlike the manual annotation technique applied in this study, the researchers used sentiment labels based on emoji lexicons to label the tweets and restricted their sentiment study to only positive or negative categories. Then they applied the N-gram for feature extractions and Naïve Bayes model for sentiment analysis. On a total of 20,827 tweets, the model had an accuracy of 89%. The researchers then used the model to study general opinions on the seven closure and suspension decisions. All except one appeared to have drawn more positive responses than negative. 

Table 1 shows a summary of the literature articles discussed above, highlighting the main algorithm used, dataset size, feature extraction methods, and accuracy achieved.

It can be noted that no previous research considered analysing tweets that are related to the topic of distance learning in Saudi Arabia. Moreover, in our research we collected and used a large dataset size with over 14,000 tweets to build a classification model, achieving excellent accuracy of 89.9%, then applied the model to study the sentiment. 

The main contributions of this research are:

1. Collecting a Twitter dataset in Arabic relating to the region of Saudi Arabia on distance learning during COVID-19 and labelling them (positive, negative).
2. Building models that can analyze the sentiment of people’s acceptance of distance learning, which feature different feature extraction approaches and different machine learning classification algorithms.
3. Conducting extensive comparative studies on the performance of the different models and selecting the model with the highest accuracy. The selected model achieved 0.899 accuracy using a combination of N-gram with Term Frequency-Inverse Document Frequency (TF-IDF) for feature extraction as well as the Logistic Regression as classification algorithm.
4. Applying the selected model on a new unlabelled dataset classified to different educational stages and concluding that, in general, positive opinions regarding distance learning for general education stages (kindergarten, intermediate, and high schools), and negative opinions at the university stage.
5. Further analysis was applied to identify the main topics related to the positive and negative sentiment, which demonstrates that negative sentiment was related to the fear of infection by making final exams in-person, the schools’ rules, and platform glitches. On the other hand, general school students demonstrate satisfaction related to the Madrasati Platform, which is the main learning platform adopted by schools in all general educational stages. This result can be used by the Ministry of Education to further improve the distance learning system.

The rest of this paper is structured as follows: Section 2 presents the research methodology. Section 3 demonstrates and discusses the experimental results and performance evaluation. Section 4 concludes and highlights the main contributions of this paper and discusses ideas for further research.

## 2. Materials and Methods

**Figure 1:**
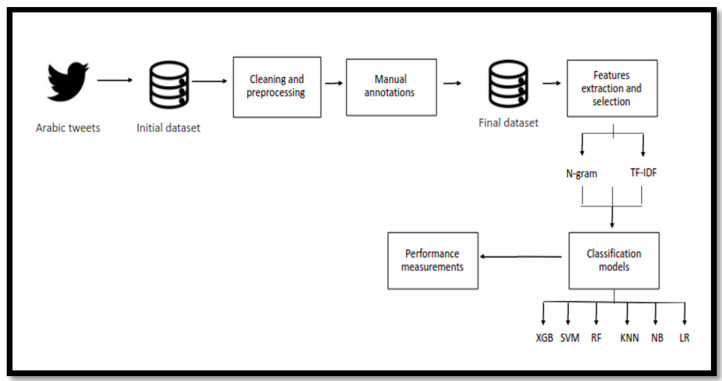
Research methodology.

The methodology followed in our research involved several stages, as depicted in Figure 1. The following subsections describe these stages in detail.

### 2.1. Dataset Collection and Description

There are different twitter scraping APIs that vary in terms of capability, including the widely used API Tweepy [12]. Tweepy is a Python library for retrieving tweets by accessing the twitter API. To use Tweepy, the Twitter user is required to apply for developer access. In addition to scraping tweets, the library can accomplish various other tasks. Retrieving tweets with Tweepy has some limitations based on account tier. The Standard Search API is restricted to tweets posted in the previous 7 days, with a limit of 3200 retrieved tweets in a timeline. Twint [13] is an open-source Python library that is designed to collect tweets. It is a free tool that can be set up easily with no need for a Twitter account or the setting up of a Twitter API and it can collect almost all available tweets. As is the case with Tweepy, Twint can collect data based on keywords, hashtags, dates, and location [14]. This research used the Twint tool with a combination of keywords and hashtags to collect two datasets of Arabic tweets about people’s opinions on distance learning at each educational stage in Saudi Arabia.

The data gathered have a significant role to play in ML, as it enables the model to learn patterns, make decisions, and perform other applications. Supervised learning requires a dataset where the label is mapped to the input feature [15]. In the experiments carried out in this research, the tweets of the first dataset were labelled (positive, negative) at the manual annotation stage and divided into the following parts:

1. Training set: contained a dataset where each input is linked with the accurate label; this allows the model to perform the learning process through training and building the model.
2. Testing set: contained unseen data that was separate from the data used in the training phase; the label is predicted by the model that was built. The predictions were then compared with the real labels to evaluate the model and calculate its accuracy.

The second dataset (application dataset) contained unlabelled data and was used to measure people’s opinions of each educational stage of distance learning, using the model that achieved the highest accuracy with the first dataset.

The same setting for downloading Arabic tweets in the region of Saudi Arabia was applied for both datasets, utilizing the Twint attributes for setting the language and the region of the tweet. Each tweet in the first dataset was then labelled either positive or negative in the manual annotation stage. The first dataset was collected from 8 March 2020 to 17 August 2020 and consisted of around 70,000 tweets. The second dataset was collected during the academic year 2020/2021 which was conducted following distance learning, specifically from 18 August 2020 to 27 April 2021, and consisted of around 92,000 tweets. Table 2 shows samples of the keywords and hashtags used for data collection.

### 2.2. Cleaning and Pre-Processing

The tweets collected for sentiment analysis are texts in a human format consisting of unstructured language, slang words, abbreviations, and orthographic mistakes. They need to be transformed to a structured format by applying some pre-processing techniques to enable the machine learning model to analyze the texts and produce reliable results with high accuracy. Therefore, pre-processing is an essential phase in natural language processing and comprises several stages based on the nature of the language and the purpose of analysis.

Analyzing Arabic tweets presents more challenges than analyzing tweets in other languages. Challenges include inconsistency of spelling and the lack of capitalization, which is necessary to identify text features. The Arabic natural language also lacks the robust tools and resources that help extract Arabic sentiments from the text. Dealing with Saudi dialects that do not follow Modern Standard Arabic (MSA) is also a challenge given their unformal grammatical structures [16,17]. 

The following steps explain the data preprocessing steps that were applied to the two datasets:

1. Removing irrelevant tweets manually that contained ads or were not related to the topic of distance learning in Saudi Arabia. Following this step, the number of tweets in the first dataset was reduced to 5096 tweets and the number in the second dataset was reduced to 9160 tweets.
2. Removing non-Arabic letters.
3. Removing symbols denoting emotions including emoticons, symbols, numbers, and hashtag sign.
4. Removing URLs and user mentions.
5. Removing Tashkeel, such as Tashdid “
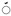
”, Fatha “
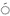
”, Tanwin Fatha “
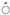
”, and Tatweel, which uses the symbol “-” to increase the length of some characters.
6. Removing punctuation.
7. Removing repeated characters, such as “طلاااااب”, which is replaced with “طلاب”.
8. Removing stop words: The stop words removed included the standard set of Arabic stop words provided by the Python’s NLTK library.
9. Applying Arabic normalization (i.e., the unification of some characters with more than one form); this step is demonstrated in Table 3.
10. Applying word stemming using ISRI stemmer to reduce Arabic words to their word stem.
11. Applying tokenization, which divides the text into smaller pieces or tokens.

**Figure 2:**
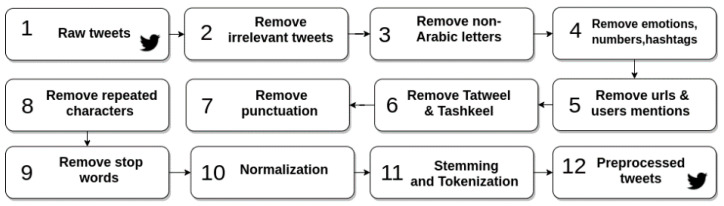
Pre-processing steps.

Table 4 demonstrates examples of implementing the pre-processing steps on a tweet, displaying the effect of each stage. Figure 2 presents a summary of the pre-processing steps applied in this research.

### 2.3. Manual Annotation

This stage was applied to the first dataset (training/testing dataset), where each tweet was manually labelled positive (1) or negative (0). The annotation guidelines used were the following:

1. Positive: If the tweet expressed positive sentiment and agreement about distance learning, it was labelled positive (1).
2. Negative: If the tweet expressed negative sentiment and disagreement about distance learning, it was labelled negative (0).

The result of this stage was: 1740 positive tweets and 3356 negative tweets.

### 2.4. Features Extraction and Selection

Natural language text cannot directly be processed by ML algorithms because text data is not computable. Therefore, a features extraction approach is implemented to convert text data into numerical vectors that the algorithms can process and work with. N-gram and the Term Frequency/Inverse Document Frequency (TF-IDF) are the most used feature extraction approaches [18].

1. N-Gram

The N-gram technique is used to preserve the captured words’ context. It uses a set of consecutive ordered words based on an N variable’s value. It is called unigram when N = 1, bigram when N = 2, and trigram when N = 3 [19].

2. Term Frequency/Inverse Document Frequency (TF-IDF)

TF-IDF is a numerical statistic that is used to measure the importance of each word to the corpus. It is a simple, well-known feature extraction method used in natural language processing, classification, and recommendation tasks. This model is implemented through two main steps. Firstly, the number of occurrences or Term Frequency (TF) of each word in the document, or in the case of this study, a tweet, is counted. Secondly, the frequency of each word occurring or Inverse Document Frequency (IDF) out of all documents (or tweets) is calculated. The smaller TF-IDF values represent common words in the corpus, which indicates that they are not significant. However, the larger TF-IDF values represent less frequent words in the corpus and are, therefore, significant. This method gives the discriminative words in the corpus more importance and value to contribute in the model than the non-discriminative word [20,21].

Equation (1) represents the equation to measure TF.

(1) $$\mathrm{TF}\left(w\right)=\frac{\mathrm{number}\;\mathrm{of}\;\mathrm{the}\;\mathrm{word}’\;s\;\mathrm{occurence}}{\mathrm{total}\;\mathrm{number}\;\mathrm{of}\;\mathrm{the}\;\mathrm{words}\in \mathrm{the}\;\mathrm{document}}$$

Equation (2) represents the equation to calculate IDF.

(2) $$\mathrm{IDF}\left(w\right)=\log\left(\frac{\mathrm{number}\;\mathrm{of}\;\mathrm{documents}}{\mathrm{number}\;\mathrm{of}\;\mathrm{documents}\;\mathrm{that}\;\mathrm{contain}\;\mathrm{the}\;\mathrm{word}}\right)$$

Then, to calculate the TF-IDF, we simply multiply the TF with IDF values for each word. The TF only aims to count the words’ occurrences in the data, where all the words have equal weight. On the other hand, the IDF provides weights based on the word’s uniqueness and importance, where common words have a lower weight than uncommon words. 

In this research, we analyzed the effect of using TF-IDF combined with N-gram as a text representation technique. The unigram with TF-IDF, and bigram with TF-IDF models, were built. The results were then fed into the machine learning algorithms. 

### 2.5. Classification Models

Classification is the task of assigning each input to a specific label or class. The use of machine learning in classification tasks helps to find similarities between instances that belong to the same class. Then, the machine can classify new instances to a certain class based on the training data. In this study, the classification task was to assign the tweet to either positive or negative using the classification models described below.

1. Support Vector Machine (SVM)

The Support Vector Machine (SVM) plots the labelled dataset on a plane to separate the classes by a hyperplane. It chooses the best hyperplane that best divides the dataset into distinct categories. When new data input is plotted above the hyperplane, it is assigned to a specific class, otherwise it is assigned to another class. There are several kernels used with the SVM algorithm, such as the linear and Radial Basis Function (RBF) kernels. The linear kernel builds linear boundaries, and it can be used whenever there are many features generated from the dataset that are applicable to text classifications. The RBF kernel is used with datasets that cannot be linearly separated; it is slower when compared to the linear kernel and is far more complicated [22]. 

Equation (3) represents the equation used for prediction in a linear kernel, using the dot product between the new input (*x*) and each support vector (*x_i_*). Equation (4) represents the RBF function, where ||*X*_1_ − *X*_2_|| is the Euclidean distance between *X*_1_ and *X*_2_, and *γ* is Gamma which is only used for the RBF kernel. SVM implements different parameters to control and enhance the model performance, such as *C*, kernel, and degree. For this experiment, *C* was set to 1 with the RBF kernel and a degree of 3.

(3) $$K\left(x,x_{i}\right)=\sum^{\;}\left(x*x_{i}\right)$$

(4) $$K\left(X_{1},X_{2}\right)=exponent\left(-\gamma {\left|\left|X_{1}-X_{2}\right|\right|}^{2}\right)$$

2. Random Forest (RF)

Random Forest (RF) is an algorithm that creates many classification decision trees. Whenever new input data needs to be classified, it will go through all the trees in the forest. Each tree will give that data a class, then the class with the highest occurrence will be chosen as the predicted class for the input data. When implementing Random Forest for data classification, *Entropy* is used to decide how to branch the nodes of the decision tree. Equation (5) represents the Entropy formula, which uses the probability of a certain outcome to determine how a node should branch. The number of estimators used with this model was set to 100.

(5) $$Entropy=\overset{C}{\underset{i=1}{\sum }}-p_{i}*log_{2}\left(p_{i}\right)$$

3. K-nearest Neighbors (KNN)

K-nearest Neighbors (KNN) assumes that similar data are close to one another. It calculates the similarities between data by some commonly used similarity measures. When new input data needs to be classified, KNN calculates the distance between the new input data and the K number of neighbors. Then, it assigns the new input data to the same class of the closest K neighbor. 

Equation (6) represents the Euclidean distance, which is usually used in KNN classification problems to calculate the centroids based on the mean between data points in a cluster. Multiple values of K neighbors were tested, and the model demonstrated the best results with 5 neighbors.

(6) $$d\left(p,q\right)=d\left(q,p\right)=\sqrt{\overset{n}{\underset{i=1}{\sum }}{\left(q_{i}-p_{i}\right)}^{2}}$$

4. Naïve Bayes (NB)

Naïve Bayes (NB) predicts the new input data class by calculating the probability using the Bayes theorem. It calculates the probabilities of each class in the dataset given the features of the input data. The class that scores the highest probability for given input data will be assigned as a class for the new input. 

Equation (7) represents the Bayes theorem, where *A* represents a class and *B* represents data.

(7) $$P(A|B)=\frac{P(B|A)\cdot P\left(A\right)}{P\left(B\right)}$$

5. Logistic Regression (LR)

Logistic Regression (LR) predicts the class of the input data based on probabilities. It uses an equation similar to linear regression to predict the class of the input data. It then uses the sigmoid function to map the predicted values to probabilities, following which it assigns a threshold value that divides the dataset into two classes. If the probability of the new input data lies above the threshold value it will be assigned to class 1; if not, it will be assigned to class 0.

Equation (8) represents the logistic function, also called the sigmoid function, where *x* is the actual value, and *e* is Euler’s number. Equation (9) represents a Logistic Regression formula, where *y* represents the predicted value, *b*_0_ is the intercept, and *b*_1_ is the coefficient of the input value *x*, which is a constant real value learned from the training data. To enhance the performance of the model, we used the following settings: Penalty = l2, Tol = 0.001, and C = 1.0.

(8) $$S\left(x\right)=\frac{1}{1+e^{-x}}$$

(9) $$y=\frac{e^{\left(b_{0}+b_{1}x\right)}}{1+e^{\left(b_{0}+b_{1}x\right)}}$$

6. XGBoost (XGB)

Extreme gradient boosting, also known as XGBoost, is a quick and scalable ML classifier. The algorithm has shown good results in challenging ML problems. It is based on the gradient tree boosting technique. It functions in parallel manners and is known for high performance and scalability. It has different parameters that could be tuned and optimized. XGB enhances its performance by applying ensemble methods in its boosting part. The ‘gradient’ part refers to the fact that it enhances future models based on previous errors. To enhance the performance of the model, we used the following settings: max_depth = 6, min_child_weight = 1, and subsample = 1.

All the classification algorithms discussed above were implemented in this research, as discussed in the results section. 

### 2.6. Performance Measurements

The Classification Accuracy corresponds to the ratio of the number of correctly classified observations to the total number of input observations. The Confusion Matrix gives a matrix as output that summarizes the performance of the model. In the Confusion Matrix, there are four important terms demonstrated in Table 5: True Positives (TP), which represents the number of tweets that are predicted as positive and are correctly positive; the True Negatives (TN) are the number of tweets that are predicted as negative and are correctly negative; the False Positives (FP) represent the tweets that are predicted as positive, but are correctly negative;, and the False Negatives (FN) are the tweets that are predicted as negative, but are correctly positive. The Confusion Matrix demonstrated in Table 5 reveals how the classification model is confused when it is making predictions. 

The accuracy of the matrix is calculated by taking the ratio of correctly classified instances over the total number of classified instances, as Equation (10) demonstrates. In our experiments, we used the measure of accuracy to evaluate the models’ performance.

(10) $$\mathrm{Accuracy}=\frac{\mathrm{TP}+\mathrm{TN}}{\mathrm{TP}+\mathrm{TN}+\mathrm{FP}+\mathrm{FN}}$$

In addition to the accuracy, we also measured the precision by calculating the false positives of the classifier recall by calculating the false negatives of the classifier and F1-score, by taking the weighted harmonic average of the recall and precision. These three measures are demonstrated by the Equations (11), (12), and (13), respectively.

(11) $$\mathrm{Precision}=\frac{\mathrm{TP}}{\left(\mathrm{TP}+\mathrm{FP}\right)}$$

(12) $$\mathrm{Recall}=\frac{\mathrm{TP}}{\left(\mathrm{TP}+\mathrm{FN}\right)}$$

(13) $$F1-\mathrm{Score}=\frac{2\times \mathrm{Precision}\times \mathrm{Recall}}{\mathrm{Precision}+\mathrm{Recall}}$$

## 3. Results

This section presents the details of the experiments conducted in this research, describing the setup of the experiments and the results of the models applied in all the experiments. It also presents a discussion on their performance.

### 3.1. Experimental Setup

All experiments were conducted using a MacBook with a 1.1 GHz Dual-Core Intel Core M processor and 8 GB 1600 MHz DDR3 memory. In terms of data splitting, in all the experiments that were conducted, the first labelled dataset was divided into 80% for training and 20% for testing. 

In this research, the randomized under-sampling technique [23,24] was applied to the first dataset in order to balance the positive and negative classes of the dataset, as the negative class was considerably higher. As a result, the total number of the tweets in the dataset was reduced to 3480 tweets, consisting of 1740 positive tweets and 1740 negative tweets.

We applied the two feature extraction approaches (N-gram and TF-IDF) discussed in Section 2.4, as well as the six classification algorithms (LR, NB, KNN, XGB, SVM, and RF) discussed in Section 2.5. 

### 3.2. The Result of Classification Models

This section summarizes the results obtained from all of the conducted experiments. The experiments were conducted over the balanced dataset. Different sizes of N-gram (unigram and bigram) in combination with the TF-IDF approach were applied to extract the features, and evaluated in conjunction with six classification algorithms (LR,NB,KNN,XGB,SVM,RF).

**Figure 3:**
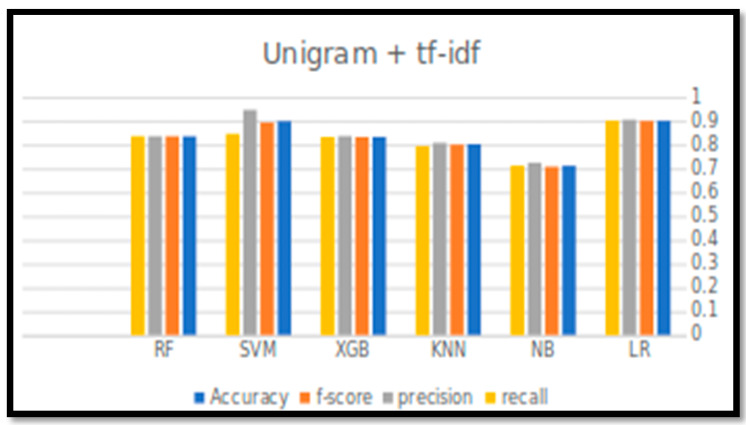
Performance measure results for all classification algorithms with unigram TF-IDF.

**Figure 4:**
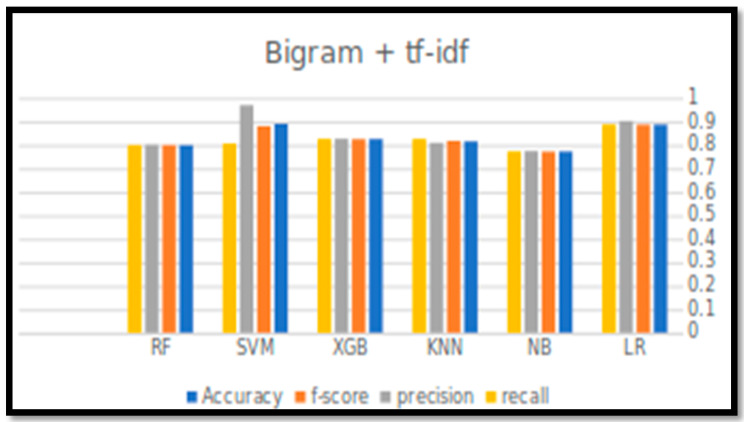
Performance measure results for all classification algorithms with bigram TF-IDF.

Table 6 and Figure 3 and Figure 4, below, indicate the output of the experiments (four performance parameters: Accuracy, Precision, Recall, and F-score) for all six classification algorithms in predicting the tweets’ sentiments.

For accuracy, it can be observed that the accuracy rate is generally decreased by increasing the size of N-gram. LR with unigram TF-IDF has achieved the best accuracy, F-score, and recall. However, the best precision value was achieved by SVM with bigram TF-IDF, with a value of 0.968. 

Although the SVM showed high performance results with unigram TF-IDF, LR performance was better, more consistent, and reliable, as reflected by all the performance measures. The accuracy for all other classifiers was above the 0.8, except the NB, which had the worst performance.

To conclude, the model that was based on the LR classifier was applied to the application dataset in order to draw conclusions about the acceptance rate of distance learning in Saudi Arabia. 

### 3.3. The Result of Sentiment Analysis

**Figure 5:**
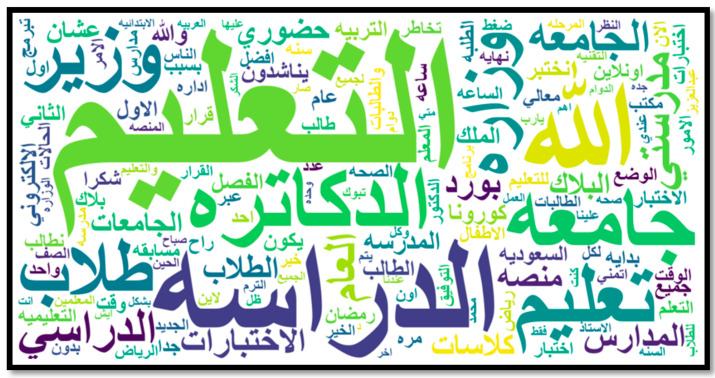
Word Cloud of common words in the application dataset.

In this section, we discuss the sentiment analysis results regarding distance learning in Saudi Arabia. We built this analysis based on the second dataset, which was pre-processed following the same steps as the first dataset. Then, we classified all tweets according to the following educational stages: primary schools and kindergarten, intermediate and high schools, or university. If the educational stage was not clear in the tweet, the tweet was classified ‘unspecified’. Table 7 demonstrates the number of tweets relating to each educational stage for the second dataset (application dataset). Figure 5 presents the most frequent words in the dataset, as generated by WordClaud library. Words shown in the figure include education, studying, exams, universities, Ministry, Blackboard, platform, Corona, and students.

We conducted the sentiment analysis using the application dataset to measure people’s opinions regarding distance learning for all educational stages, applying the LR model with unigram and TF-IDF as a feature extraction approach. 

**Figure 6:**
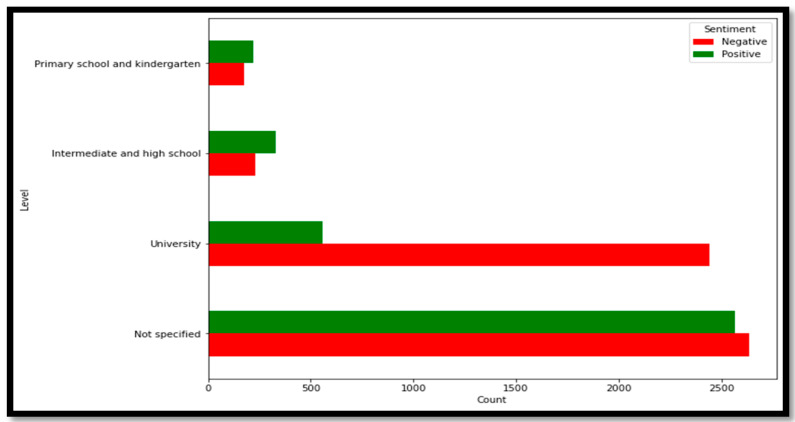
The opinions regarding distance learning in Saudi Arabia for different educational stages.

The analysis in Figure 6 shows that the university stage tweets had the highest percentage of negative sentiment regarding distance learning, while the primary school and kindergarten, intermediate, and high schools’ tweets had the highest positive reaction. In terms of tweets that were not clearly related to specific educational stages, the sentiment was slightly toward the negative side.

We then investigated the dominating sentiment for each educational stage and analysed the top 20 frequent words.

**Figure 7:**
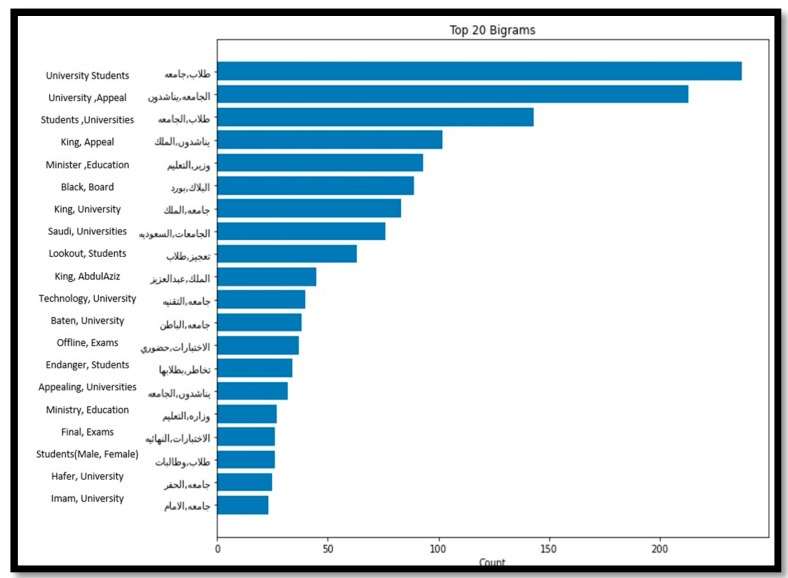
Top 20 Frequent words from the university’s negative tweets.

Looking closely at university stage tweets, the main reasons for the negative sentiments were related mainly to the final exams and the decisions of some universities to hold exams in-person. Students were expressing negative sentiments toward holding final exams on campus due to their fear of getting infected, which was demonstrated clearly by analysing the top 20 frequent words of the negative tweets related to universities. As Figure 7 shows, words repeated in this set of tweets include final exams, exams attendance, request king, and universities, highlighting the main concern of students was to request holding exams online. However, universities who conducted their final exams on campus took into consideration all the precautionary measures, which resulted in successful completion of final exams without any reported infection cases.

**Figure 8:**
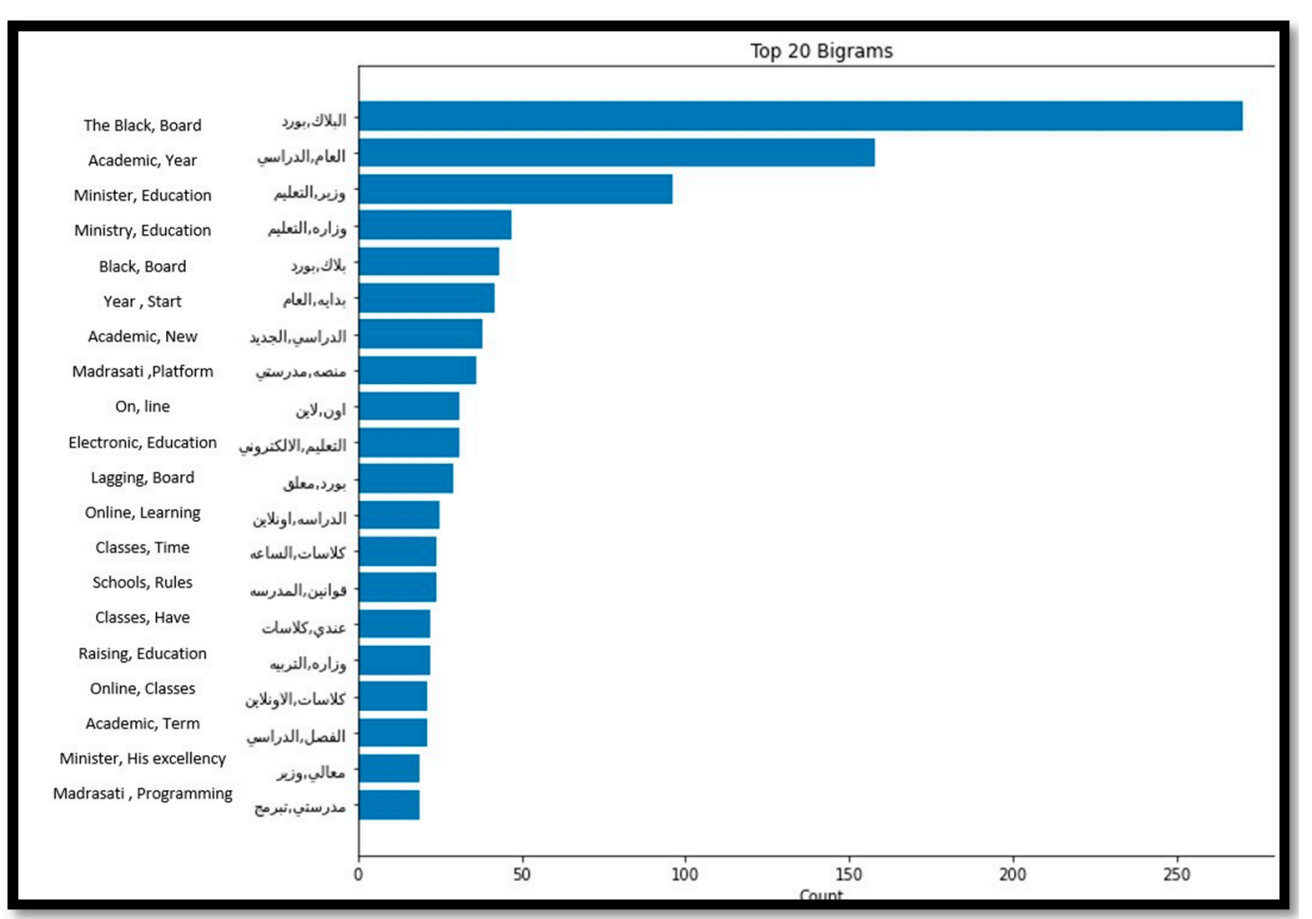
Top 20 Frequent words from the unspecified negative tweets.

Regarding the top 20 frequent words for the negative tweets of the unspecified educational stages, the main concerns were related to Blackboard glitching, Madrasati platform issues, and school rules, as indicated in Figure 8. 

**Figure 9:**
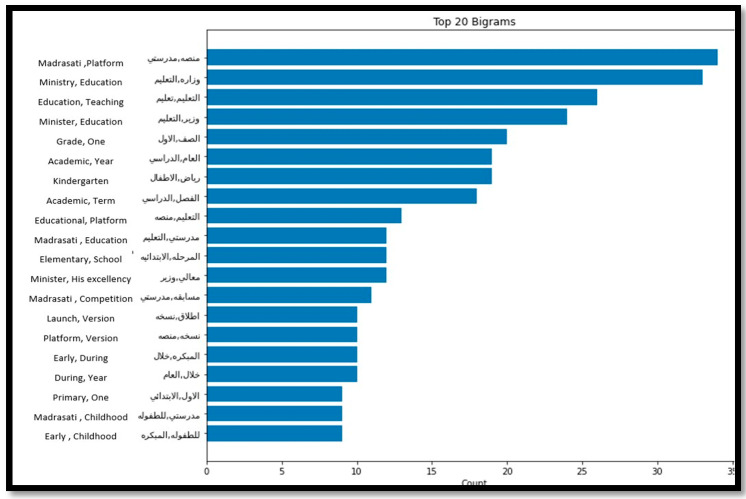
Top 20 Frequent words from the primary schools’ and kindergartens’ positive tweets.

**Figure 10:**
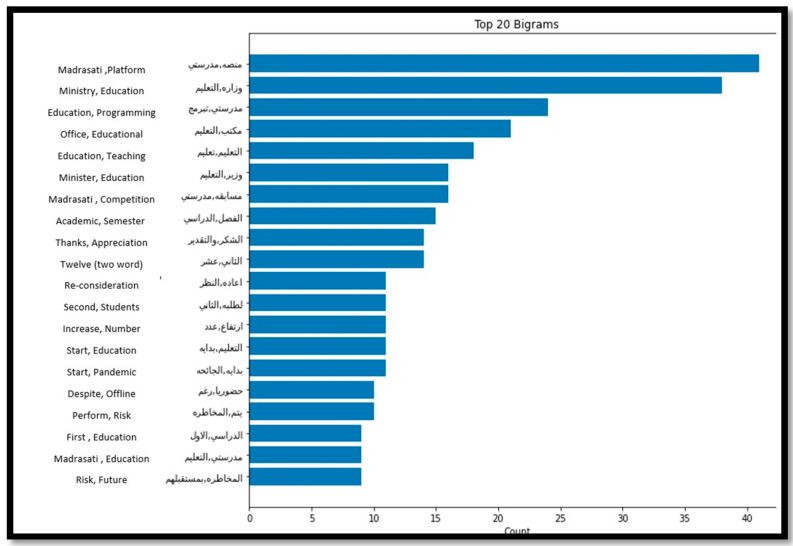
Top 20 Frequent words from the intermediate and high schools’ positive tweets.

On the other hand, analysing the top 20 frequent words for the positive tweets related to primary, kindergarten, intermediate, and high school educational stages, demonstrated by Figure 9 and Figure 10, reveal that people were talking positively about the Ministry of Education and Madrasati Platform. The platform was developed by The Ministry of Education for general education students as an interactive educational distance learning platform. It contributed greatly to the continuation of the educational process during the COVID-19 pandemic by offering various educational services and activities.

**Figure 11:**
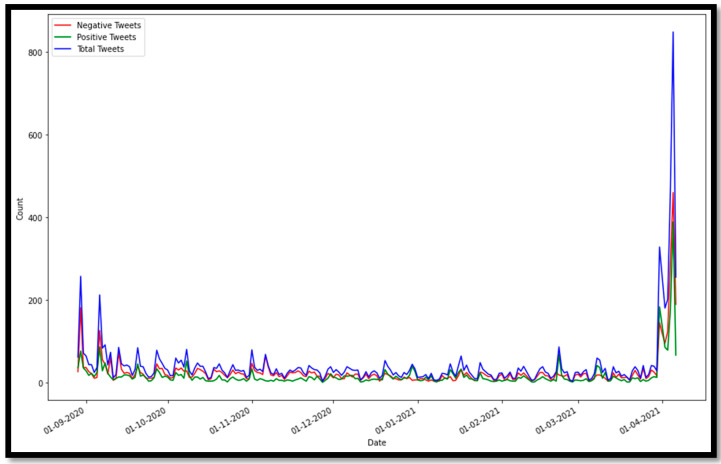
Time series analysis of all tweets in application dataset.

Moreover, we investigated the change of sentiments for all educational stages overtime since the beginning of the academic year, which is demonstrated by Figure 11, below.

It can be observed that the first and the last month of the academic year have an increase in the number of tweets among Twitter users. At the beginning of the year, students were not sure if they were going to continue the learning process following the distance learning approach, resulting in many discussions being raised around this topic. Whereas, at the end of the year, students targeted social media platforms to express their feelings about their exams, grades, and graduation. Negative sentiment tweets have exceeded the positive sentiments throughout the whole year, except for some time periods such as the beginning of March 2021 when the Saudi Arabia government announced that students will continue studying through the distance learning approach to the end of the academic year. Noticeably, November and December 2020 displayed the least positive sentiments because students have their first semester finals in this period, and due to the uncertainty of how second semester will be conducted.

## 4. Discussion

This rapidly expanding pandemic that is threatening the health system is a global concern and a serious challenge to public health systems and governments worldwide. Countries, including Saudi Arabia, are adopting different actions and prevention measures to control the spread of the virus. One such measure is the shifting of the education system for all educational stages to distance learning. Despite the Ministry’s efforts to facilitate distance learning, various challenges have surfaced due to the sudden transition. 

This research provides the design and implementation of Arabic tweet sentiment analysis regarding distance learning in Saudi Arabia. The tweets collected were posted within the period of March 2020 and April 2021 within the region of Saudi Arabia. Two different datasets were collected and pre-processed. The first dataset was manually labelled according to two categories: positive or negative and used to train and test the accuracy of the model. Different sentiment analysis models were built by exploiting N-gram and TF-IDF for features extraction and six different classification algorithms. The models were then applied to the first dataset to identify the model that yielded the highest accuracy level. The highest accuracy achieved was 0.899, using a model that comprised a unigram and TF-IDF as the feature extraction approach, and LR as the machine learning classification algorithm. 

This model was then applied to the second dataset, which was not labelled and was used to gain insight into the opinion of distance learning at all educational stages in Saudi Arabia. The results demonstrated generally positive opinions regarding distance learning for general education stages (kindergarten, intermediate, and high schools), and negative opinions at the university stage. Further analysis was applied to identify the main topics related to the positive and negative sentiment. 

The results of our study could assist the Ministry of Education to assess the distance learning limitations, such as platform glitching and the distance learning school rules employed and investigate ways to improve the service.

In the future, we aim to collect new data to study the sentiment regarding other COVID-19 related issues, such as COVID vaccines and their side effects. Moreover, we aim to investigate and apply more optimisation techniques to the pre-processing stage, such as applying some frameworks [25] to handle negation words for Arabic language, and to study their effect on the level of accuracy obtained. Furthermore, we may conduct comparative performance analysis after applying different sets of deep learning models, as well as testing of new transformer-based models for language representation, such as BERT [26], which was developed by Google, and has recently been pre-trained for the Arabic language [27,28] and proved to be efficient in different natural language processing domains, including sentiment analysis. The region of the study may also be widened to cover different areas and countries as well as to study the effect of other COVID-19 prevention measures.

## Figures and Tables

**Table 1:** Summary of related research applied models and accuracy.

Reference	Dataset Size (Number of Tweets)	Classifier	Feature Extraction	Accuracy
[5]	2026	SVM	Unigram, Bigram, TF-IDF	91%
[6]	2000	Logistic Model Trees	Information Gain measure	85.5%
[7]	1633	RF	BoW and TF-IDF.	91%
[8]	1121	SVM	TF-IDF and N-grams	84.84%
[9]	6122	Liblinear	BoW	87.5%
[11]	20,827	NB	N-gram	89%

**Table 2:** Samples of the keywords and hashtags used for data collection.

Educational Stage	Example of Keywords Used	Example of Hashtags Used
University	الجامعة اونلاين، البلاكبورد، دوام الجامعة، طلاب جامعة، المحاضرات عن بعد، جامعة، جامعي، الجامعيين، رجعونا حضوري، نبي حضوري Online university, Blackboard, University hours, University students, Online lectures, University, Attended education, we want attended education.	متضررين_جامعة_الامام_عن_بعد #اونلاين_جامعة_الملك_سعود # جامعة_طيبه_نبى_تقويم_مستمر# التعليم_الجامعي# نبغى_حضوري_للجامعيين_والثانوي# جامعة_الأميرة_نورة# #Imam_Univirsity_Aggrieved_ٍStudents #Online_Saud_University #Taiba_Univisity_Demand_Continuous_Assessments # University_Education #We_Want_Attended_Education_High_School_and_University #Prince_Norah_University
Intermediate/High schools	الطلاب عن بعد، التعلم عن بعد، الدراسة اونلاين، التعليم الالكتروني، الغاء النهائي، الغاء الترم، كورونا، طلاب الابتدائي، طلاب الثانوي، طلاب الثانوية، طالبات المتوسطة، المنصة، منصتي، المعلين، المعلمات، متعب عن بعد، المدارس Students at Distance learning, Distance Education, Online learning, Electronic learning, Cancel the finals, End the semester, COVID-19, Elementary schools’ students, High schools’ students, Intermediate schools’ students, Schools, Madrasati platform, Teachers, Difficult distance learning, Schools	#نطالب_بإلغاء_العام_الدراسي # رياض_الأطفال # الروضة_الإفتراضية # تأجيل_الدراسة # نطالب_بالدراسه_حضوري # المعلمين_والمعلمات# #Stop_ Academic _Year #Kindergarten #Virtual_Kindergarten #Postpone_Schools #Teachers #We_Demand_Attended_Education
Primary schools/kindergarten		

**Table 3:** Arabic text normalization.

Letter	After Normalization
ى	ي
ؤ or ئ	ء
ة	ه
أ or إ or آ	ا

**Table 4:** Example of implementing the pre-processing steps on a tweet.

Raw Tweet	ودي صراحه آخر ترم يكون حضوووري أودع الجامعة 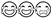 #التعليم_عن_بعد
After removing hashtags, symbols, punctuation, and numbers	ودي صراحه آخر ترم يكون حضوووري أودع الجامعة التعليم عن بعد
After removing Tashkeel, Tatweel, and repeated characters	ودي صراحه آخر ترم يكون حضوري أودع الجامعة التعليم عن بعد
After removing stop words	ودي صراحه آخر ترم يكون حضوري أودع الجامعة التعليم بعد
After normalization	ودي صراحه اخر ترم يكون حضوري اودع الجامعة التعليم بعد
After stemming	ودي صرح اخر ترم يكون حضر ودع جمع علم بعد
After tokenization	[‘ودي’, ‘صرح’, ‘اخر’, ‘ترم’, ‘يكون’, ‘حضر’, ‘ودع’, ‘جمع’, ‘علم’, ‘بعد’]

**Table 5:** The Confusion Matrix.

	Positive	Negative
Positive	TP	FP
Negative	FN	TN

**Table 6:** Performance results from using TF-IDF extraction with different N-grams for all classification algorithms.

	Classifier	Accuracy	F-Score	Precision	Recall
**Unigram + tf-idf**	**LR**	0.899	0.899	0.904	0.899
	**NB**	0.711	0.707	0.723	0.711
	**KNN**	0.801	0.800	0.807	0.793
	**XGB**	0.831	0.831	0.835	0.831
	**SVM**	0.897	0.892	0.945	0.844
	**RF**	0.834	0.834	0.834	0.834
**Bigram + tf-idf**	**LR**	0.886	0.885	0.900	0.886
	**NB**	0.771	0.771	0.772	0.771
	**KNN**	0.814	0.816	0.808	0.824
	**XGB**	0.824	0.824	0.825	0.824
	**SVM**	0.889	0.879	0.968	0.804
	**RF**	0.798	0.798	0.799	0.798

**Table 7:** Number of tweets in each educational stage for the second dataset (application dataset).

	Primary Schools and Kindergarten	Intermediate and High School	University	Unspecified
Total no. of tweets	396	560	3000	5203

## Data Availability

The data presented in this study are available upon request from the corresponding author. The data are not publicly available, as this research is ongoing.

## References

[1] Worldometer (2021). COVID-19 Coronavirus Pandemic. https://www.worldometers.info/coronavirus/.

[2] Cumulative Cases. https://coronavirus.jhu.edu/data/cumulative-cases.

[3] Education in Saudi Arabia. https://wenr.wes.org/2020/04/education-in-saudi-arabia.

[4] (2020). MOE Leading Efforts to Combat COVID-19 Pandamic. https://iite.unesco.org/wp-content/uploads/2020/10/The-Saudi-MOE-Leading-Efforts-to-Combat-Coronavirus-Pandemic-COVID-19.pdf.

[5] Alayba A.M., Palade V., England M., Iqbal R. Arabic language sentiment analysis on health services. Proceedings of the 2017 1st international workshop on arabic script analysis and recognition (asar).

[6] Aloqaily A., Al-Hassan M., Salah K., Elshqeirat B., Almashagbah M. (2020). Sentiment analysis for Arabic tweets datasets: Lexicon-based and machine learning approaches. J. Theor. Appl. Inf. Technol..

[7] Aljarah I., Habib M., Hijazi N., Faris H., Qaddoura R., Hammo B., Abushariah M., Alfawareh M. (2020). Intelligent detection of hate speech in Arabic social network: A machine learning approach. J. Inf. Sci..

[8] Al-Rubaiee H., Qiu R., Alomar K., Li D. (2016). Sentiment Analysis of Arabic Tweets in e-Learning. J. Comput. Sci..

[9] Almouzini S., Khemakhem M., Alageel A. (2019). Detecting Arabic Depressed Users from Twitter Data. Procedia Comput. Sci..

[10] Fan R.E., Chang K.W., Hsieh C.J., Wang X.R., Lin C.J. (2008). LIBLINEAR: A library for large linear classification. J. Mach. Learn. Res..

[11] Alhajji M., Al Khalifah A., Aljubran M., Alkhalifah M. (2020). Sentiment analysis of tweets in Saudi Arabia regarding governmental preventive measures to contain COVID-19. Preprints.

[12] tweepy.api—Twitter API Wrapper. https://docs.tweepy.org/en/latest/api.html.

[13] Pratama A. (2020). How to Scrape Tweets from Twitter with Python Twint. https://medium.com/analytics-vidhya/how-to-scrape-tweets-from-twitter-with-python-twint-83b4c70c5536.

[14] Hwang J. (2020). What Python Package Is Best for Getting Data from Twitter? Comparing Tweepy and Twint. https://towardsdatascience.com/what-python-package-is-best-for-getting-data-from-twitter-comparing-tweepy-and-twint-f481005eccc9.

[15] Osisanwo F.Y., Akinsola J.E., Awodele O., Hinmikaiye J.O., Olakanmi O., Akinjobi J. (2017). Supervised Machine Learning Algorithms: Classification and Comparison. Int. J. Comput. Trends Technol..

[16] Nassr Z., Sael N., Benabbou F. (2020). Preprocessing Arabic dialect for sentiment mining: State of art. ISPRS-Int. Arch. Photogramm. Remote. Sens. Spat. Inf. Sci..

[17] Alwakid G., Osman T., Hughes-Roberts T. (2017). Challenges in Sentiment Analysis for Arabic Social Networks. Procedia Comput. Sci..

[18] Rahman S.S.M.M., Biplob K.B.M.B., Rahman H., Sarker K., Islam T. (2020). An Investigation and Evaluation of N-Gram, TF-IDF and Ensemble Methods in Sentiment Classification. International Conference on Cyber Security and Computer Science.

[19] Kowsari K., Meimandi K.J., Heidarysafa M., Mendu S., Barnes L., Brown D. (2019). Text Classification Algorithms: A Survey. Information.

[20] Dhar A., Dash N.S., Roy K. (2018). Application of TF-IDF Feature for Categorizing Documents of Online Bangla Web Text Corpus. Intelligent Engineering Informatics.

[21] Qaiser S., Ali R. (2018). Text Mining: Use of TF-IDF to Examine the Relevance of Words to Documents. Int. J. Comput. Appl..

[22] Raschka S. (2016). How to Select Support Vector Machine Kernels. https://www.kdnuggets.com/2016/06/select-support-vector-machine-kernels.html.

[23] Mishra S. (2017). Handling Imbalanced Data: SMOTE vs. Random Undersampling. Int. Res. J. Eng. Technol..

[24] Hasanin T., Khoshgoftaar T. The Effects of Random Undersampling with Simulated Class Imbalance for Big Data. Proceedings of the 2018 IEEE International Conference on Information Reuse and Integration (IRI).

[25] Duwairi R.M., Alshboul M.A. Negation-Aware Framework for Sentiment Analysis in Arabic Reviews. Proceedings of the 2015 3rd International Conference on Future Internet of Things and Cloud.

[26] Devlin J., Chang M.W., Lee K., Toutanova K. (2018). BERT: Pre-training of deep bidirectional transformers for language understanding. arXiv.

[27] Antoun W., Baly F., Hajj H. (2020). AraBERT: Transformer-based Model for Arabic Language Understanding. arXiv.

[28] Abdul-Mageed M., Elmadany A., Nagoudi E.M.B. (2020). Arbert & Marbert: Deep Bidirectional Transformers for Arabic. arXiv.

